# Acute phase reactants roles in the early diagnosis of preterm prelabour rupture of membranes: A case-control study

**DOI:** 10.1097/MD.0000000000045913

**Published:** 2025-11-14

**Authors:** Yusuf Ziya Kizildemir, Veysel Toprak, Orhan Yanar

**Affiliations:** a Department of Obstetrics and Gynecology, Şanliurfa Training and Research Hospital, Şanliurfa, Turkey; b Department of Obstetrics and Gynecology, Private Metrolife Hospital, Şanliurfa, Turkey; c Department of Obstetrics and Gynecology, Private Nev Hospital, Şanliurfa, Turkey.

**Keywords:** biomarkers, diagnostic tests, premature prelabour rupture of membranes

## Abstract

Early diagnosis of premature membrane rupture is important to reduce the risk of feto-maternal complications. The aim of this study was to evaluate the role of serum acute phase reactants in the early diagnosis of premature prelabour rupture of membranes (PPROM). In this case-control study, the role of acute phase reactants in the early diagnosis of PPROM was evaluated in 60 pregnant women with PPROM compared to 60 healthy pregnant women. It was found that mean albumin and factor 12 values were lower, while C-reactive protein (CRP), sedimentation, ceruloplasmin, fibrinogen and protein S values were higher in patients with PPROM. In the classification of PPROM, the areas under the curve (AUC) and cutoff values were determined: albumin (AUC = 0.735, cutoff < 36 g/dL), factor 12 (AUC = 0.732, cutoff < 207.4%), CRP (AUC = 0.698, cutoff > 4.3 mg/L), ferritin (AUC = 0.660, cutoff > 10.39 ng/mL), ceruloplasmin (AUC = 0.788, cutoff > 0.44 mg/dL), fibrinogen (AUC = 0.764, cutoff > 3.74 mg/dL), protein S (AUC = 0.656, cutoff > 42%). In the logistic regression analysis to predict the probability of PPROM according to the optimal cutoff values, the risk of PPROM was found to be OR (odds ratio) = 6.69 times higher in patients with albumin < 36 g/dL, OR = 8.5 times higher in patients with ferritin > 10.39 ng/mL, OR = 4.66 times higher in patients with ceruloplasmin > 0.44 mg/dL, OR = 3.19 times higher in patients with fibrinogen > 3.74 mg/dL and OR = 3.64 times higher in patients with protein S > 42%. The sensitivity of the logistic regression model was found to be 88.3% with a specificity of 81.7%. The likelihood ratio (LR)(+) value of the model was 4.82, and LR(−) value was 0.14. In the Fagan nomogram, if the model was positive, the probability of developing PPROM in the patient was calculated as 67.4%, and if the model was negative, the probability of not developing PPROM was calculated as 87.5%. The combined use of acute phase reactants in the early diagnosis of PPROM may be a helpful diagnostic tool in suspected cases.

## 1. Introduction

Prelabour rupture of membranes (PROM) is the rupture of the amniotic sac before the onset of labor. If it occurs before the 37th week of gestation, it is called “preterm prelabour rupture of membranes (PPROM)” and occurs in approximately 2% of pregnancies.^[1,2]^ Several clinical factors may help to predict the risk of PPROM, like a history of PPROM, the presence of pathogens in the vagina or cervical fluid, or the presence of histologically diagnosed chorioamnionitis, which may lead to intrauterine infections.^[3,4]^ Increased levels of proinflammatory cytokines are also reported as a cause of PPROM in the absence of an infection.^[5]^ PPROM may lead to neonatal sepsis, and long-term neurodevelopmental disorders and may require neonatal intensive care unit admission.^[6]^ PPROM is diagnosed by cervical examination. However, in cases where the diagnosis is unclear, additional tests such as detecting oligohydramnios by ultrasonography (USG) or blood tests may help clinicians.^[7]^ The diagnosis may also be provided by some nonspecific biomarker tests, but they are less commonly used as their value in the diagnosis of a membrane rupture is limited as they mostly indicate a decidual deterioration. Following the microbial invasion of the amniotic fluid, inflammatory processes may develop in the gestational sac, fetal membranes, chorion decidua, fetus and various maternal compartments. Therefore, a maternal serum biomarker may significantly help clinical decision-making, such as immediate delivery of the fetus.^[8–12]^ Early diagnosis is important to reduce the risk of foeto-maternal complications.^[13]^

The aim of this study was to evaluate the role of serum acute phase reactants (i.e., C-reactive protein, erythrocyte sedimentation rate (ESR), ferritin, haptoglobin, ceruloplasmin, fibrinogen, albumin, transferrin, alpha-fetoprotein, factor 12, and protein S) in the early diagnosis of PROM in pregnant patients followed up with suspicion PROM in the obstetrics and gynecology clinic.

## 2. Materials and methods

### 2.1. Research type and sampling

This observational case-control study was conducted between 2023 and 2024 on pregnant patients who were followed up for PPROM in the Gynaecology and Obstetrics Clinic of Şanliurfa Training and Research Hospital, Şanliurfa, Turkiye (clinical trial number: not applicable). All patients first applied to the emergency department and were admitted to the clinics with a preliminary diagnosis. Permission for the study was obtained from the Clinical Research Ethics Committee of Faculty of Medicine Harran University, Şanliurfa, Türkiye. The study was conducted in accordance with the guidelines of the Declaration of Helsinki and approved by the Ethics Committee of Clinic Studies of Faculty of Medicine, Şanliurfa University (Approval No: HRÜ/23.08.29), with protocol code 227703 and approval date May 8, 2023. The minimum sample size was calculated as 102 people with a power of 80%, type 1 error of 0.05 and *d* of 0.1. Considering any loss in follow-up, the sample was increased by approximately 20% and the study was completed with 120 people.

### 2.2. Groups

The case group consisted of pregnant women between 24th and 34th weeks of gestation who were hospitalized in the clinic with suspicion of PROM, consisting of patients who applied within the date range of the study and who verbal consent to voluntarily participate after being informed about the study.

The control group included 60 healthy pregnant women between 24th and 34th weeks of gestation, who applied to the clinic for routine control and gave informed consent for voluntarily participating in the study. Age, gestational week and the absence of any health problem were taken into consideration in matching the control group with the case group.

In frequency matching, patients and controls were taken in a 1:1 ratio.

Informed consent forms were obtained from all case and control groups.

### 2.3. Inclusion and exclusion criteria

- Pregnant women between 24th and 34th gestational weeks were included.
- Women with a history of antepartum hemorrhage, high-risk pregnancies (multiple pregnancies, polyhydramnios, gestational diabetes mellitus, etc), patients with chronic diseases (hypertension, autoimmunity, thrombophilia, diabetes, thyroid diseases, etc), pregnant women with acute or chronic infectious diseases were excluded from the study.

## 3. Measurements

### 3.1. Acute phase reactants

C-reactive protein (CRP), ESR, ferritin, haptoglobin, ceruloplasmin, fibrinogen, albumin, transferrin, factor 12 and alpha-fetoprotein (AFP) levels were obtained and analyzed at the time of admission in both the patient and control groups. Acute phase reactants are inexpensive and easily accessible tests in clinical settings.

### 3.2. Vaginal culture

A vaginal culture is performed to a sample of discharge, aspirated endocervical and endometrial fluids and cervical swabs. The test aims to detect bacteria and/or fungi that may be the source of infection causing abnormal vaginal discharge, vaginal or pelvic pain or irregular bleeding. The samples were taken from all pregnant women in both the patient and control groups.

### 3.3. Urine culture

Urine culture is a laboratory test used for the diagnosis and treatment of urinary tract infections. Urine samples were collected in a sterile container and inoculated on specific media. The results were evaluated as “growth” or “no growth.”

### 3.4. Statistical analysis

SPSS®20 (IBM Corp., Armonk) and JAMOVI® (ver. 2.3.28) softwares were used for data analysis. Numerical data were presented as mean, standard deviation and percentage. Kolmogorov–Smirnov test was preferred for the evaluation of normal distribution. Chi-square test, Student *t* test, receiver operating characteristic (ROC) analysis, binary logistic regression model and Fagan Nomogram were used in the analyses. ROC analysis was performed to determine the place of albumin, transferrin, AFP, factor 12, CRP, ESR, ferritin, haptoglobin, ceruloplasmin, fibrinogen, and protein S levels in the classification of PPROM. The Youden index was used to find the optimal cutoff point. The classification was made according to the determined cutoff points and a logistic regression model was created to predict the probability of PPROM based on these values. Post-test probability ratios of the logistic regression model were calculated with the Fagan nomogram. A *P* < .05 was considered statistically significant.

## 4. Results

The mean age of the pregnant women included in the study was 28.65 ± 7.34 years (min. 18 − max. 49). It was found that mean albumin and factor 12 values were statistically significantly lower and CRP, ESR, ceruloplasmin, fibrinogen and protein S values were significantly higher in patients with PPROM. No significant difference was found between the vaginal flora and urine culture growth results between the groups (Table 1).

**Table 1:** Comparison of acute phase reactant levels and demographic characteristics in the case and control groups.

Parameters	Groups		*P*	Cohen *d*
	PPROM n = 60	Control n = 60		
	Mean ± SD or n (%)	Mean ± SD or n (%)		
Age (yr)	29,15 ± 7.35	28.15 ± 7.35	.458	
Gestational week (wk)	24.35 ± 4.56	24.55 ± 4.77	.890	
Albumin (g/dL)	34.38 ± 3.56	37.38 ± 3.56	**<.001**	0.84
Transferrin (mg/dL)	383.92 ± 67.06	398.92 ± 67.06	.223	0.22
AFP (ng/mL)	196.98 ± 122.44	207.28 ± 122.44	.646	0.08
Factor 12 (%)	180.82 ± 40.23	198.02 ± 40.23	**.021**	0.42
CRP (mg/L)	12.93 ± 15.96	3.17 ± 2.13	**<.001**	0.85
ESR (mm/h)	34.73 ± 17.8	28.73 ± 17.88	**.038**	0.33
Ferritin (ng/mL)	21.87 ± 19.81	17.77 ± 19.81	.259	0.20
Haptoglobin (mg/dL)	1.14 ± 0.41	1.02 ± 0.41	.119	0.28
Ceruloplasmin (mg/dL)	0.45 ± 0.08	0.35 ± 0.08	**<.001**	1.16
Fibrinogen (mg/dL)	4.27 ± 1.13	3.17 ± 1.13	**<.001**	0.96
Protein S (%)	40.25** ± **13.78	32.25** ± **13.78	**.002**	0.58
Vaginal flora culture result (+)	6 (10)	5 (8.3)	.751	
Urine culture result (+)	4 (6.6)	3 (5)	.696	

Values in bold indicate statistical significance (*P* < .05). AFP = alpha-fetoprotein, CRP = C-reactive protein, ESR = erythrocyte sedimentation rate, PPROM = premature prelabour rupture of membranes, SD = standard deviation.

**Figure 1.**
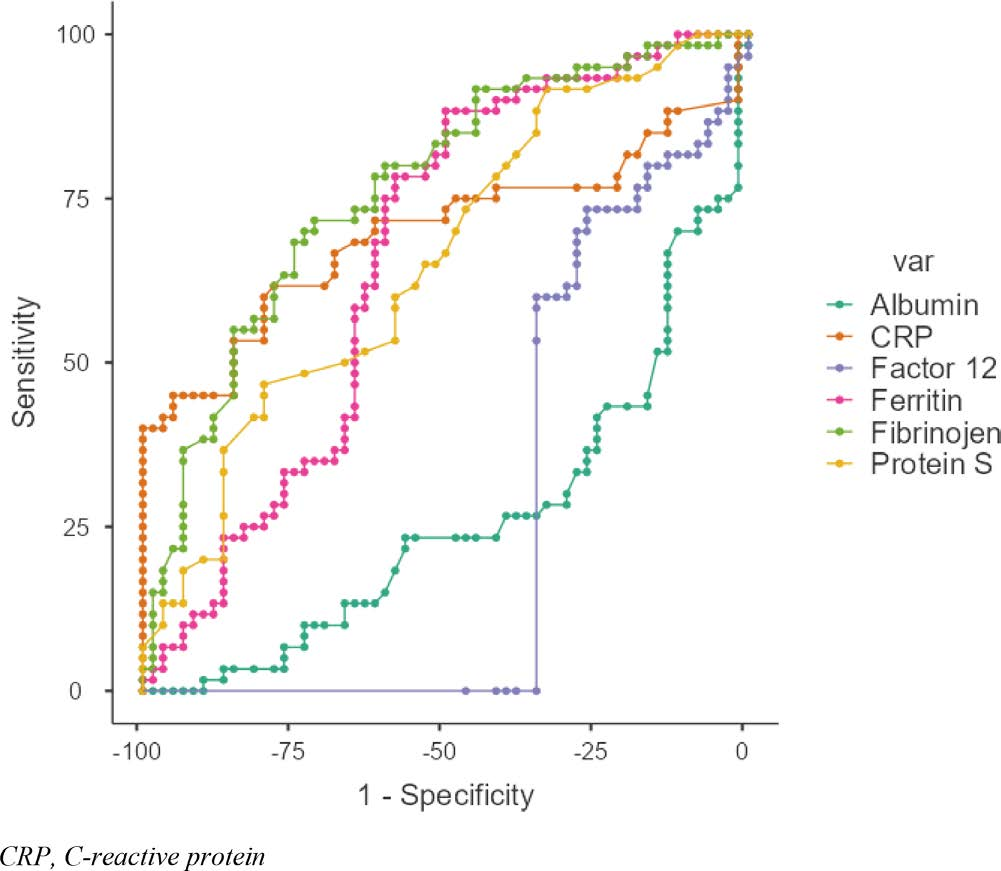
Areas under the curve for acute phase reactants. CRP = C-reactive protein.

The place of acute phase reactants in the classification of PPROM was evaluated by ROC analysis (Fig 1.). It was found that the area under the curve was significant for albumin, factor 12, CRP, ferritin, ceruloplasmin, fibrinogen, and protein S; while not significant for other reactants. In the classification of PPROM, ceruloplasmin, fibrinogen, albumin and factor 12 tests were found to be tests of moderate power; while CRP, ferritin, and protein S tests were found to be tests of weak power. It was found that values below the optimum cutoff value for albumin and factor 12 and values above the optimum cutoff value for CRP, ferritin, ceruloplasmin, fibrinogen and protein S were diagnostic for PPROM. The optimal cutoff values found in our study for all acute phase reactants are given in Table 2.

**Table 2:** ROC analysis results for acute phase reactants and optimal cutoff values.

Acute phase reactants	Optimal cutoff	Sensitivity (%)	Specificity (%)	PPV (%)	NPV (%)	Youden index	AUC	*P*
Albumin (g/dL)	<36	71.67	70.00	70.49	71.19	0.417	0.735	<.001
Factor 12 (%)	<207.4	100.00	65.00	74.07	100.00	0.650	0.732	<.001
C-reactive protein (mg/L)	>4.3	61.67	78.33	74	67.14	0.400	0.698	<.001
Ferritin (ng/mL)	>10.39	88.33	50.00	63.86	81.08	0.383	0.660	.003
Ceruloplasmin (mg/dL)	>0.44	61.67	81.67	77.08	68.06	0.433	0.788	<.001
Fibrinogen (mg/dL)	>3.74	71.67	71.67	71.67	71.67	0.433	0.764	<.001
Protein S (%)	>42	46.67	80.00	70	60	0.267	0.656	.003

AUC *=* area under the curve, NPV *=* negative predictive value, PPV *=* positive predictive value, ROC = receiver operating characteristic.

**Figure 2.**
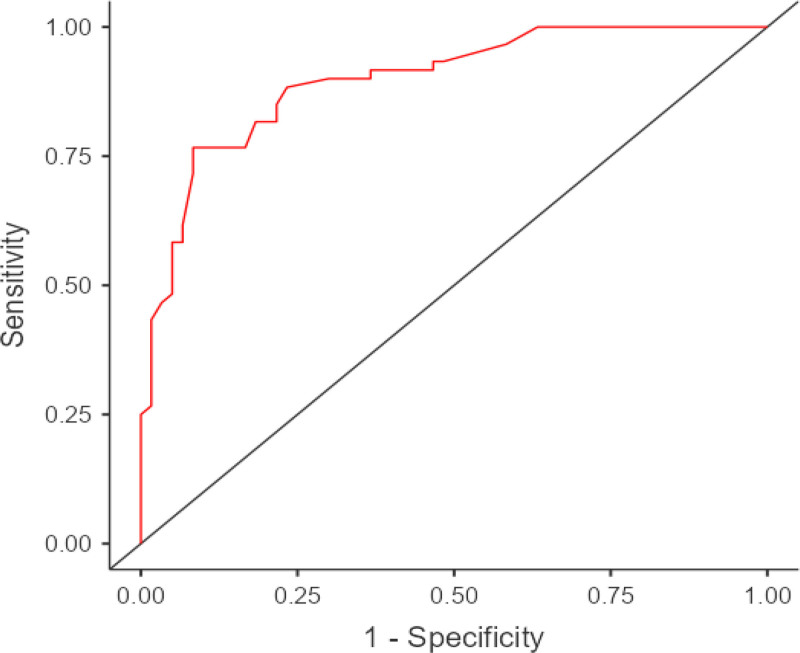
The area under the curve for the logistic regression model.

The patient and control groups were classified according to the cutoff values found in the study and Forward likelihood ratio (LR) binary logistic regression analysis was performed to predict the probability (risk) of PPROM. Variables with significant areas under the curve (albumin, factor 12, CRP, ferritin, ceruloplasmin, fibrinogen, and protein S) were included in the model. Risk categories were determined according to the cutoff values given in Table 2. It was found that the model was significant (omnibus test *P* < .001), Nagelkerke R Square value was 0.574 and the model fit was adequate. The accuracy rate of the model was found to be 85%, sensitivity 88.3% and specificity 81.7%. Model performance was evaluated by ROC analysis and the area under the curve for the model was found to be 0.897 (Fig. 2), therefore the model was evaluated to show a strong performance.

Among the variables included in the model, albumin, ferritin, ceruloplasmin, fibrinogen, and protein S were found to significantly contribute to the model. The risk of PPROM was found to be 6.69 times higher in patients with albumin < 36 g/dL, 8.5 times higher in patients with ferritin > 10.39 ng/mL, 4.66 times higher in patients with ceruloplasmin > 0.44 mg/dL, 3.19 times higher in patients with fibrinogen > 3.74 mg/dL, and 3.64 times higher in patients with protein S > 42% (Table 3).

**Table 3:** Binary logistic regression analysis for PPROM risk prediction.

Variables	*B*	*P*	OR	95% CI for OR	
				Lower	Upper
Albumin					
Risk category < 36 g/dL)	1.901	<.001	6.69	2.38	18.80
Ferritin					
Risk category > 10.39 (ng/mL)	2.141	<.001	8.50	2.65	27.23
Ceruloplasmin					
Risk category > 0.44 (mg/dL)	1.540	.013	4.66	1.39	15.63
Fibrinogen					
Risk category > 3.74 (mg/dL)	1.160	.046	3.19	1.02	9.96
Protein S					
Risk category > 42 (%)	1.293	.021	3.64	1.21	10.94
Constant	0.055	.862	1.05		

CI = confidence interval, OR = odds ratio, PPROM = premature prelabour rupture of membranes.

The probability formula for PPROM obtained as a result of logistic regression is as follows;


$$\begin{array}{l} p=\frac{e^{0.055+1.901Albumin+2.141Ferritin+1.293Protein\;\;\;S+1.160Fibrinogen}}{1+e^{0.055+1.901Albumin+2.141Ferritin+1.293Protein\;\;\;S+1.160Fibrinogen}} \end{array}$$

Note: For each variable in the formula, the calculation should be made by writing 1 if it is over the cutoff value and 0 (zero) if below.

The estimation results of the model are given in Table 4. Post-test probabilities were calculated for the prediction values of the model with the Fagan nomogram (Table 5).

**Table 4:** Regression model classification results.

Observed	Predicted		
	Groups		Percentage correct
	Control	PPROM	
Groups			
Control	49	11	81.7
PPROM	7	53	88.3
Overall percentage			85.0

PPROM = premature prelabour rupture of membranes.

**Table 5:** Likelihood ratios for the model.

	Ratios
Post-test disease probability	67.4%
Post-test health probability	94.2%
Positive likelihood ratio	4.82
Negative likelihood ratio	0.14

**Figure 3.**
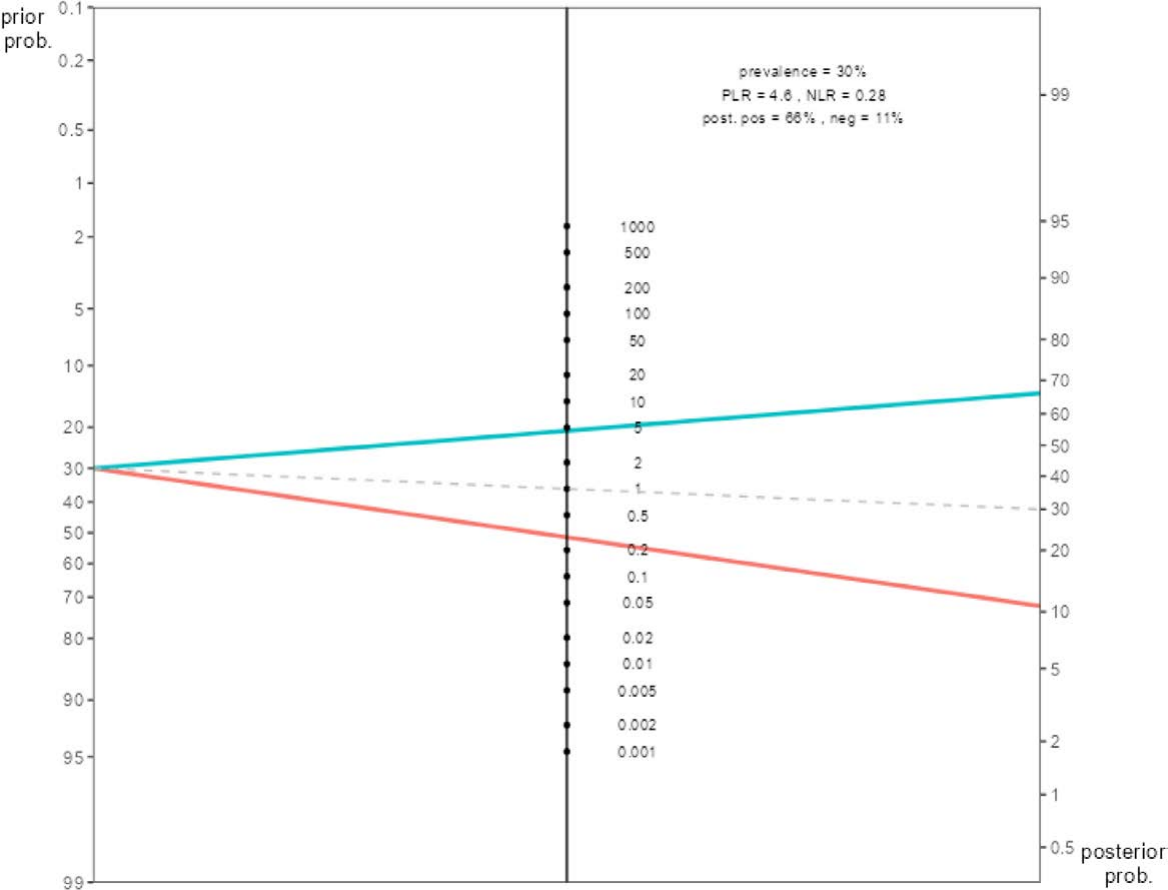
Fagan nomogram. In the nomogram, the values on the left show the pretest probability; the line in the middle shows the likelihood ratios (the green top line crosses over the positive likelihood value, and the red bottom line crosses over the negative likelihood value); and the values on the right show the post-test probability. neg = post-test NLR, NLR = negative likelihood ratio, PLR = positive likelihood ratio, post. pos = post-test positive likelihood ratio, posterior prob = post-test probability, prior prob = pretest probability.

Considering the post-test probability ratios, if the model resulted positively, the probability of developing PPROM in the patient was calculated as 67.4%, and if the model resulted negatively, the probability of not developing PPROM was calculated as 87.5% (Table 5). In other words, a positive model result for a patient will increase our prediction of having PPROM to 67% (Table 5, Fig. 3). For this model, the positive LR was calculated as 4.82, while the negative LR as 0.14 (Table 5).

## 5. Discussion

Due to the risk of preterm labor, accurate prediction of PPROM is a very important issue. At this point, biomarkers can be used to predict the risk of PPROM. Biomarkers usually include proteins, hormones or other molecules that can be measured in maternal serum, amniotic fluid or urine test. This study aimed to predict PPROM based on biomarkers measured in maternal serum. In the ROC analysis performed for the classification of PPROM, it was found that ceruloplasmin, fibrinogen, albumin and factor 12 tests were moderate power tests in PPROM classification, while CRP, ferritin, and protein S tests were weak power tests. According to the cutoff values we found in our study, the sensitivity of the logistic regression analysis to predict the probability of PPROM was found to be 88.3% with a specificity of 81.7%. Among the variables included in the model, albumin, ferritin, ceruloplasmin, fibrinogen, protein S made a significant contribution to the model and the risk of PPROM was found to be 6.69 times higher in patients with albumin < 36 g/dL, 8.5 times higher in patients with ferritin > 10.39 ng/mL, 4.66 times higher in patients with ceruloplasmin > 0.44 mg/dL, 3.19 times higher in patients with fibrinogen > 3.74 mg/dL, and 3.64 times higher in patients with protein S > 42%. The post-test probability ratios of the regression model were calculated as 67.4% if the model was positive and 87.5% if the model was negative. In other words, a positive model result for a patient would increase our prediction that the patient had PPROM to 67%. In this case, the result reaches the diagnostic threshold (50%). We can conclude that PPROM would develop in cases when the regression model yielded a positive classification (LR(+) = 4.82, LR(−) = 0.14).

In the literature, there are studies evaluating the predictive value of various biomarkers for PPROM. Wang et al found that placental protein 14 (PP14) had excellent diagnostic accuracy for PPROM, with a sensitivity of 100% and a specificity of 87.5% for a cutoff value of 0.008 mcg/mL. It was suggested that PP14 might be a new potential biomarker for PPROM and might be converted into a bedside test to rapidly diagnose PPROM.^[14]^ Kahouadji et al found that chemokine C-X-C motif ligand 1 (CX3CL1) level measured in the first trimester was significantly increased in PPROM cases compared with controls and predicted PPROM with a sensitivity of 90% and a specificity of approximately 40%. The combination of CX3CL1 and maternal risk factors significantly improved the area under the curve. CX3CL1 has been reported as a promising blood biomarker in the prediction of PPROM in the first trimester.^[15]^ El-Achi et al reported that biomarkers measured during first-trimester screenings like pregnancy-associated plasma protein-A (PAPP-A), and βHCG did not predict PPROM. The area under the ROC curve for the prediction model was 0.67 and expressed moderate effectiveness.^[16]^ Chiu et al found that low levels of maternal serum placental growth factor (PlGF) and PAPP-A in the first trimester were associated with an increased risk of PPROM in pregnant women < 37 gestational weeks. However, it was reported that additional biomarkers and further research were needed to improve the screening performance of the combined model which would include maternal risk factors, PAPP-A and PlGF before any clinical application.^[17]^ In the study conducted by Rosen et al, it was found that circulating maternal plasma thrombin-antithrombin (TAT) complexes predicted PPROM and the probability of PPROM was 6.0 times higher in patients with TAT level > 3.9 mcg/L in the second trimester. For this cutoff value, sensitivity was reported to be 88%, specificity 68%, positive predictive value 82% and negative predictive value 97%.^[18]^ Buyuk et al reported that maternal serum haptoglobin levels were higher in the PPROM group in the second and third trimesters of pregnancy in patients presenting with PPROM, with an optimum cutoff of 94.5 mg/dL for haptoglobin and an 80% of sensitivity and specificity for the diagnosis of PPROM.^[19]^ In the study conducted by Nergiz Avcioğlu et al, mean s-Endoglin levels in the PPROM group were found to be lower than in the control group, and interleukin-6 (IL-6), white blood cell counts and CRP levels were found to be higher in the PPROM group. No difference was found between the ROC analysis results of s-Endoglin in predicting preterm delivery in the PPROM group.^[20]^ In the study by Uçkan et al, it was found that subclinical inflammatory factors, which were known to be closely related to inflammation, were not associated with PPROM, but were associated with unfavorable postpartum outcomes.^[21]^ Kim et al found that several inflammatory biomarkers (IL-6/8, M-CSF, MMP-8 and TNFR2 were found to be high in cervicovaginal fluid in women with PPROM.^[22]^ Karakus and Dogan investigated the relationship between maternal serum amino acid levels and PPROM and found that lysine, glycine and glutamic acid levels were significantly higher in the PPROM group compared to the control group. Lysine had the highest predictive accuracy with a threshold value exceeding 137.90 μmol/L, an area under the curve of 0.796 (*P* < .001), a sensitivity of 66.7% and a specificity of 80.0%. Glycine and glutamic acid were also found to effectively predict the development of PPROM.^[23]^ PPROM risks were estimated in studies and different results were reported. It was observed that studies in which multiple markers were used produced better prediction results compared to studies in which a single biomarker was used. In our study, PPROM was predicted by using a combination of a group of biomarkers and the prediction power of the logistic regression model developed was found to be high. Compared to the predictions made with a single biomarker, the predictive power was found to be better in our study where more than one biomarker was used. We can conclude that acute phase reactants measured in maternal serum may be diagnostic in suspected PPROM cases where the diagnosis of ruptured membranes is not correct based on physical examination and other diagnostic tests.

## 6. Limitations and strengths

The single-center nature of our study and the small sample size were our limitations, while the combined use of multiple biomarkers and easily accessible tests in the clinic were our strengths.

## 7. Conclusion

According to the results of our study, serum ceruloplasmin, fibrinogen, albumin and factor 12 tests were found to be tests of medium power, while CRP, ferritin, and protein S tests were found to be weak power tests in the early diagnosis of PPROM. The logistic regression-based prediction model using these tests in combination, yielded a sensitivity of 88.3% and a specificity of 81.7%. Posttest probability ratios for the regression model were calculated as 67.4% if the model resulted positive and 87.5% if the model resulted negative. The model we developed may be a helpful diagnostic tool for clinicians in suspected PPROM cases. The validity of our regression-based probability formula needs to be confirmed in prospective studies conducted in different centers and populations.

## Author contributions

**Conceptualization:** Yusuf Ziya Kizildemir, Veysel Toprak, Orhan Yanar.

**Methodology:** Yusuf Ziya Kizildemir, Veysel Toprak .

**Formal analysis :** Veysel Toprak, Yusuf Ziya Kizildemir.

**Investigation:** Veysel Toprak, Yusuf Ziya Kizildemir.

**Supervision:** Orhan Yanar.

**Writing – original draft:** Yusuf Ziya Kizildemir.

**Writing – review & editing:** Orhan Yanar, Veysel Toprak, Yusuf Ziya Kizildemir.
